# Frontal and Insular Input to the Dorsolateral Temporal Pole in Primates: Implications for Auditory Memory

**DOI:** 10.3389/fnins.2019.01099

**Published:** 2019-11-12

**Authors:** Marta Córcoles-Parada, Mar Ubero-Martínez, Richard G. M. Morris, Ricardo Insausti, Mortimer Mishkin, Mónica Muñoz-López

**Affiliations:** ^1^Human Neuroanatomy Laboratory, School of Medicine, University of Castilla-La Mancha, Albacete, Spain; ^2^Department of Anatomy, Catholic University, Murcia, Spain; ^3^Centre for Discovery Brain Sciences, University of Edinburgh, Edinburgh, United Kingdom; ^4^Laboratory of Neuropsychology, National Institute of Mental Health, Bethesda, ML, United States

**Keywords:** auditory memory, limbic memory circuit, superior temporal gyrus, frontal, insula, primate, dorsolateral temporal pole

## Abstract

The temporal pole (TP) has been involved in multiple functions from emotional and social behavior, semantic processing, memory, language in humans and epilepsy surgery, to the fronto-temporal neurodegenerative disorder (semantic) dementia. However, the role of the TP subdivisions is still unclear, in part due to the lack of quantitative data about TP connectivity. This study focuses in the dorsolateral subdivision of the TP: area 38_DL_. Area 38_DL_ main input originates in the auditory processing areas of the rostral superior temporal gyrus. Among other connections, area 38_DL_ conveys this auditory highly processed information to the entorhinal, rostral perirhinal, and posterior parahippocampal cortices, presumably for storage in long-term memory (Muñoz-López et al., 2015). However, the connections of the TP with cortical areas beyond the temporal cortex suggest that this area is part of a wider network. With the aim to quantitatively determine the topographical, laminar pattern and weighting of the lateral TP afferents from the frontal and insular cortices, we placed a total of 11 tracer injections of the fluorescent retrograde neuronal tracers Fast Blue and Diamidino Yellow at different levels of the lateral TP in rhesus monkeys. The results showed that circa 50% of the total cortical input to area 38_DL_ originates in medial frontal areas 14, 25, 32, and 24 (25%); orbitofrontal areas Pro and PAll (15%); and the agranular, parainsular and disgranular insula (10%). This study sets the anatomical bases to better understand the function of the dorsolateral division of the TP. More specifically, these results suggest that area 38_DL_ forms part of the wider limbic circuit that might contribute, among other functions, with an auditory component to multimodal memory processing.

## Introduction

The temporal pole (TP), a cortical area only present in primates, has several anatomical subdivisions with progressive changes in architecture as one moves from medial agranular limbic toward the more dorsolateral dysgranular paralimbic division. These subdivisions have distributed anatomical connections with limbic structures to neocortical regions. Due, in part, to the complexity of these connections, the TP has been involved in a great diversity of functions. While the role of the TP in emotional and social behavior is associated with its anatomical and functional connections with amygdala, rostral superior temporal gyrus (rSTG) and medial and orbitofrontal cortex (Baron-Cohen et al., 1999; Beauregard et al., 2001; Tillfors et al., 2001), the TP involvement in memory seems to be associated to its dense connections with the medial temporal cortex (Insausti et al., 1987; Suzuki and Amaral, 1994a; Muñoz-López et al., 2015). Moreover, the degeneration of the TP in fronto- temporal (semantic) dementia suggests that the TP is key as a semantic hub in cortex (Mummery et al., 2000; Hodges and Patterson, 2007; Lambon Ralph and Patterson, 2008; Acosta-Cabronero et al., 2011; Lambon Ralph et al., 2017), and human fMRI data also support its role in language (Spitsyna et al., 2006). The TP is very relevant in epilepsy surgery due to its anatomical proximity to and connectivity with the hippocampus, amygdala and adjacent cortex (Dupont et al., 2001). However, the division of labor of the TP subdivisions remains unclear, due, in part, to the lack of quantitative anatomical data on connectivity.

This work focuses in the afferent connections of the dorsolateral division of the TP and follows a series of anatomical studies aimed to understand the anatomical organization of the higher order auditory processing (Munoz-Lopez et al., 2010; Muñoz-López et al., 2015).

Area 38_DL_ of the TP is situated at the forefront of the rSTG and is involved in higher order auditory processing (Poremba et al., 2004; Gil-da-Costa et al., 2006; Poremba, 2006; Ng et al., 2014). Consistent with this function, auditory processing areas of the rSTG (i.e., Ts1, Ts2, and TAa) account for about 30% of its cortical input (Muñoz-López et al., 2015). The reciprocal connections of area 38_DL_ with the entorhinal cortex (EC), the most anterior part of the perirhinal cortex (areas 35 and 36), and the posterior parahippocampal cortex (areas TH/TF), point to this pathway as critical for auditory memory (Insausti et al., 1987; Suzuki and Amaral, 1994a; Muñoz-López et al., 2015). However, although still far from being understood, the complexity of the connections of area 38_DL_ are suggestive of a relevant role in auditory memory, but as part of a wider network and also in other cognitive functions as well.

First, the role of area 38_DL_ in memory is still unclear in part because the rSTG-38_DL_-parahippocampal-perirhinal-EC-hippocampus pathway is anatomically more restricted than the visual one. Projections from area 38_DL_ go to EC, TH/TF, and area 35 do exist, but bypass most of area 36 of the perirhinal cortex (Muñoz-López et al., 2015). In contrast, the visual TE-parahippocampal-perirhinal-EC-hippocampus projections extend to the whole of areas 35 and 36 of the perirhinal cortex (Insausti et al., 1987; Witter et al., 1989; Witter and Amaral, 1991; Suzuki and Amaral, 1994a,b). Moreover, perirhinal lesions impair visual memory (Meunier et al., 1996; Malkova et al., 2001) but leave auditory memory intact (Fritz et al., 2005). A key issue is whether the organization of the auditory memory pathway may be different from that of the visual system (Fritz et al., 2005; Munoz-Lopez et al., 2010; Muñoz-López et al., 2015).

Second, area 38_DL_ forms part other networks (Jones and Powell, 1970; Mesulam and Mufson, 1982; Mufson and Mesulam, 1982; Markowitsch et al., 1985; Morán et al., 1987; Kondo et al., 2003; Saleem et al., 2008) potentially involved in several other aspects of cognition. Furthermore, although the TP is larger and more complex in humans than in non-human primates, comparative studies indicate that they share topological and cytoarchitectonic features (Blaizot et al., 2010; Insausti, 2013), rendering non-human primate studies so valuable. In addition to the morphological similarities, the connections of the TP subdivisions in humans also resemble those seen in monkeys (Jones and Powell, 1970; Mesulam and Mufson, 1982; Mufson and Mesulam, 1982; Markowitsch et al., 1985; Morán et al., 1987; Kondo et al., 2003; Saleem et al., 2008), although with a more expanded circuitry as demonstrated by structural diffusion tensor MRI data (Fan et al., 2014). This expanded connectivity in humans is even more extensive when functional connectivity MRI is considered (Pascual et al., 2015). This has led to the hypothesis that the TP in humans is a cortical hub enabling interactions of multiple cortical areas (Lambon Ralph et al., 2017); perhaps with special emphasis in social cognition (Baron-Cohen et al., 1999; Beauregard et al., 2001; Tillfors et al., 2001) as well as in emotions (Lambon Ralph et al., 2017) and semantic cognition (Mummery et al., 2000; Hodges and Patterson, 2007; Lambon Ralph and Patterson, 2008; Acosta-Cabronero et al., 2011; Lambon Ralph et al., 2017).

It is difficult to appraise these hypotheses rigorously in the absence of quantitative data on connectivity, and this is where detailed anatomical studies with primates remain critical to establish the fundamental structural connectivity. Our previous study on the anatomical organization of the dorsolateral TP area 38_DL_ showed that, along with its major auditory afferents (30%), this input runs in parallel from various other cortical areas, such as the polysensory area of the superior temporal sulcus (TPO) accounting for about 10%, and the medial temporal cortex for an additional 10% (Muñoz-López et al., 2015). Our aim in this study was to extend our quantitatively analysis to determine the contribution of the frontal and insular cortex input to area 38_DL._ We aimed to provide a functional interpretation of the anatomical data primarily within the framework of auditory memory, but also on other cognitive functions for which the TP may play an important role, such as emotional and social behavior and semantic cognition.

With this aim, we placed small deposits of fluorescent retrograde tracer injections, Fast Blue (FB) and Diamidino Yellow (DY), at different levels of the lateral temporal pole, from dorsal to more ventral locations. We generated representative coronal sections, two-dimensional unfolded maps and histograms to illustrate the laminar distribution and density of projecting neurons.

## Materials and Methods

### Subjects

Rhesus monkeys (*Macaca mulatta, N* = 8) of both sexes weighting between 6.0 and 10.0 Kg were used. This study was based originally on four rhesus monkeys with intact brains as an extended analysis of connectivity reported in Muñoz-López et al. (2015), but to maximize data while minimizing the number of animals used, four additional rhesus monkeys (3, 6, 7, 8) that had forebrain commissurotomy before tracer injections were also used (Muñoz et al., 2009). This gave a total of 8 brains, but necessarily we focused on intact ipsilateral connectivity. This is justified because forebrain commissurotomized cases showed a similar ipsilateral pattern of distribution of labeling to that of intact brains. Experiments were carried out in strict adherence to the Guide for Care and Use of Laboratory Animals and under approved NIMH Animal Study Proposal and the European Union rules for care and use of animals (UE 86/609/CEE), and the supervision and approval of the Ethical Committee of Animal Research of the University of Castilla-La Mancha, Spain.

### Tracers

**Figure 1:**
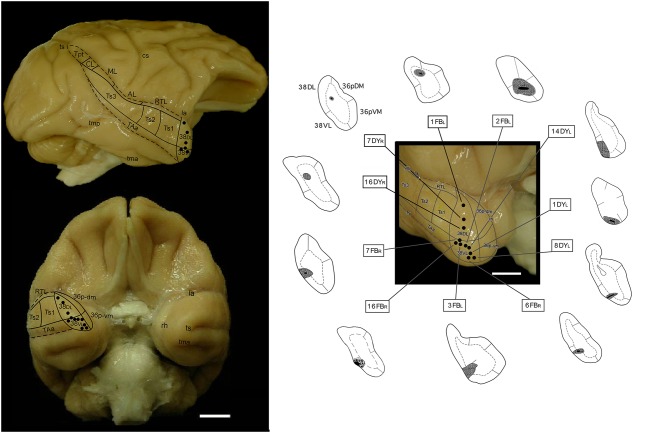
Lateral (upper) and orbital (bottom) views of the *Macaca mulatta* brain surface illustrating the approximate location of retrograde tracer injections (fast blue (FB) and diamidino yellow (DY) in the lateral temporal pole (TP). The subdivisions of the lateral TP (38_DL_ and 38_VL_), and of the medial TP (36p_DM_ and 36p_VM_), are indicated as well as the visible belt auditory areas. On the right-side panel, approximate location of the tracer injection sites. AL, anterior lateral auditory belt area; cs, central sulcus; la, lateral sulcus; ML, middle lateral auditory belt area; rh, rhinal sulcus; RTL, rostrotemporal lateral auditory belt area; TAa, superior temporal gyrus area TAa; tma, anterior middle temporal sulcus; tmp, posterior medial temporal sulcus; Tpt, temporoparietal area; ts, superior temporal sulcus; Ts1-3, superior temporal gyrus areas Ts1-Ts3.

Details are described previously (Muñoz-López et al., 2015). Briefly, 11 discrete 1 μl injections of the fluorescent retrograde tracers FB and DY (Sigma Chemical CO, St. Louis, MO), suspended in distilled water at concentrations of 3% (FB) and 2% (DY), were injected with a Hamilton syringe at a depth of 1.5–2 mm below the cortical surface (Figure 1).

### Tissue Processing

After a survival period of 2 weeks, animals were deeply anesthetized with pentobarbital and perfused transcardially with 4% paraformaldehyde as described earlier (Munoz-Lopez et al., 2010). The brains were blocked by cutting approximately the caudal 1 cm of the occipital lobe in the coronal plane, cryoprotected, and quickly frozen in isopentane (−80°C). Brains were then cut in the coronal plane at 50 μm continuously from the frontal to the occipital pole. We collected one-in-10 series. Six sections were processed for thionin, retrograde label analysis, myeloarchitectonic evaluation with Gallyas myelin stain, parvalbumin, cytochromoxidase, and acetilcholinesterase as described in previous studies (Muñoz et al., 2009; Muñoz-López et al., 2015).

### Data Analysis

Coronal sections were analyzed every 1 mm throughout the whole cerebral cortex except for the occipital pole, although we present here only data dealing with labeling in frontal and insular cortices of the ipsilateral hemispheres to the injection sites. The number of labeled neurons was counted and the distribution of retrograde labeling was plotted with an Axiophot Zeiss microscope equipped with a digital video camera (CCD, Optronics, Goleta, CA) and an image analysis system (Bioquant Nova, R&M Biometrics Inc., Nashville, TN).

Unfolded two-dimensional maps were constructed for each monkey following the procedure of Van Essen and Maunsell (1980) as reported previously (Insausti and Muñoz, 2001; Muñoz and Insausti, 2005). Briefly, lines were traced through layer IV (or in its absence the border between cortical layers III and V) in each coronal section across the frontal lobe and insular cortex. Labeled cells found in layers I-III (supragranular) were then represented at the left-hand side for each section's line (layer IV), while those in layers V-VI were represented on the right-hand side.

**Figure 2:**
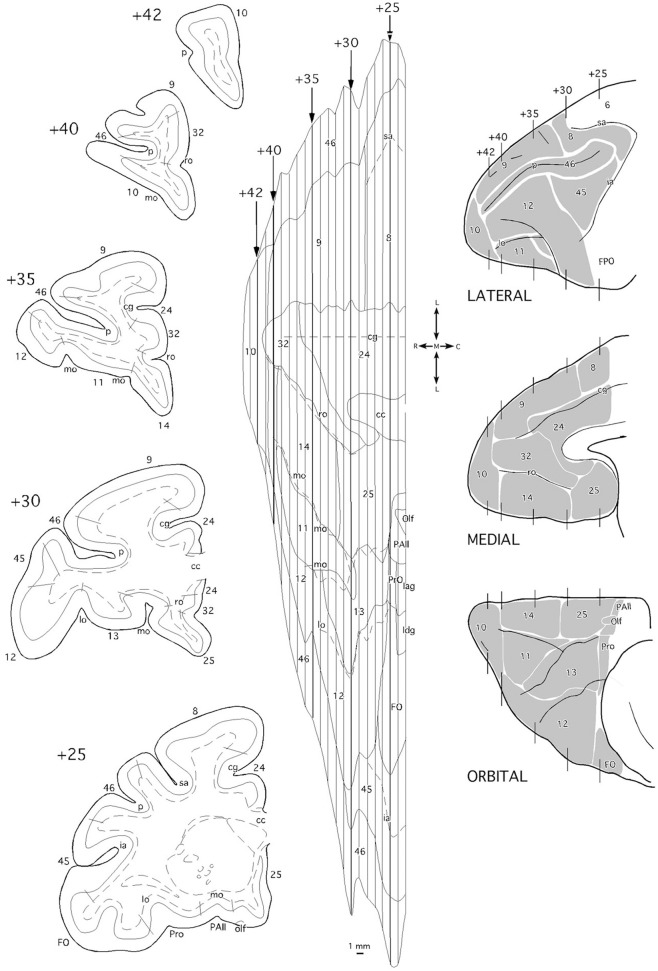
Coronal sections (left side), two-dimensional unfolded map, and lateral, medial, and orbital views (right side) of the *Macaca mulatta* brain indicating the architectonic divisions of the frontal cortex. In the unfolded map, vertical lines correspond to the position of the coronal sections from rostral (+42) to caudal (+25) in mm from the posterior commissure. Layer IV is delineated with a continuous line while the white matter boundary is represented with dashed lines in the coronal section schematic drawings. Dashed lines in the unfolded map represent the frontal sulci. cc, *corpus callosum*; cg, cingular gyrus; FO/FPO, frontal operculum; ia, inferior ramus of the arcuate sulcus; Iag, insular agranular cortex; Idg, insular disgranular cortex; lo, lateral orbital sulcus; mo, medial orbital sulcus; olf, olfactory area; p, sulcus principalis; PAll, frontal periallocortical area; Pro, frontal proisocortical area; ro, rostral sulcus; sa, superior ramus of the arcuate sulcus.

To create unfolded maps of the frontal cortex, we use the cingulate sulcus as the unfolding central reference, so it occupies the center of each frontal map (see Figure 2). Medial prefrontal, orbitofrontal, and lateral frontal cortex, including the ventral bank of the principal sulcus as far as its fundus, are represented ventral to the cingulate sulcus. Medial prefrontal and dorsolateral prefrontal cortices (areas 9 and 8) including the dorsal bank of the principal sulcus, were so above the cingulate sulcus. Thence, area 46 was represented divided into two approximate halves.

**Figure 3:**
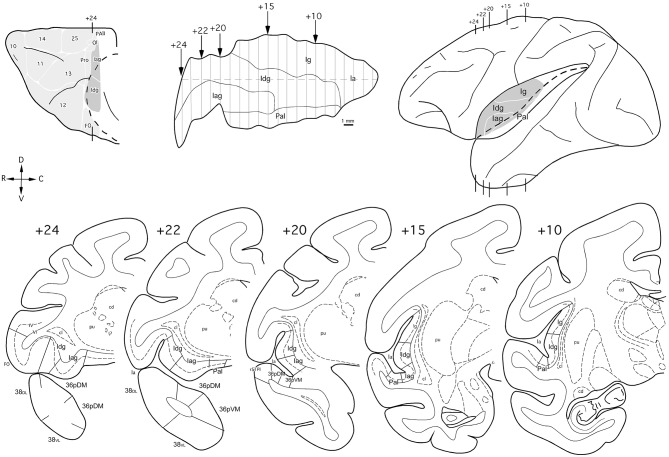
Orbital and lateral views (insular regions in gray), unfolded map, and coronal sections (bottom) through the insula in the *Macaca mulatta* brain with the cytoarchitectonic divisions used in this study. Arrows in the map and lines in the brain indicate the rostro (+24) caudal (+10) level of the coronal sections in mm from the posterior commissure. Abbreviations as in previous figures: cd: caudate nucleus; cl, *claustrum*; cs, circular sulcus; Ig, insular granular cortex; PaI, parainsula; pu, putamen.

The insular cortex was unfolded following an imaginary line through the middle of its dorsoventral extent. The insular cortical areas and the circular sulcus are represented in Figure 3.

The number of retrogradely neurons in the cerebral cortex is reported in tables as percentages as follows: (a) within each lobe and (b) out of the total number of retrogradely labeled neurons in the entire cerebral cortex (except the occipital pole) outside the TP (thus excluding intrinsic connections).

### Presentation of the Results

Results are described in the text quantitatively as a percentage of labeled neurons in each architectonic area with respect to the total number of labeled neurons within either the whole cortical label or their own area, i.e., frontal or insular cortex (see **Tables 2**, **3**). The results refer to the ipsilateral hemisphere. Tracer injection uptake is variable, and therefore, the number of labeled neurons in each case differs, even when the same volume of tracer is injected in the same architectonic area and following the same technical procedure. To take this into account, we express the density of labeling in terms of minimum-maximum percentage of labeled neurons after each individual injection when describing the results.

Given that percentages provide only a relative appreciation of the projection, we have added histograms with raw numbers of retrogradely labeled neurons in representative cases to illustrate an estimation of the density of the projection (see Figure 9). Figures with coronal sections and unfolded maps illustrate the laminar and topographical distribution of the retrograde labeling.

## Results

### Nomenclature

#### Temporal Cortex

The temporal cortex architectonic areas were delimited following the criteria of Seltzer and Pandya (1978) with some modifications to adapt the rhesus monkey terminology to the *Macaca fascicularis* terminology according with Insausti et al. (1987) and Munoz-Lopez et al. (2010).

The nomenclature for the TP has been described previously (Munoz-Lopez et al., 2010; Muñoz-López et al., 2015). Briefly, the temporopolar cortex has been divided into two divisions, medial and lateral. The latter has been subdivided into dorsal (38_DL_) and ventral (38_VL_). Area 38_DL_ is the largest and closely related with the rSTG in terms of connections and cyto- and chemoarchitecture, while area 38v_L_ is associated with the rostral superior temporal sulcus and inferotemporal cortex. There are other two divisions that occupy the medial surface of the TP and for those, we have kept the term 36p because they remind the architectonic features and connections of area 36 of the perirhinal cortex. Area 36p has been divided into a ventromedial division, 36_pVM_, and a dorsomedial one, 36_pDM_, which correspond to previous architectonic nomenclature used for this area (36pm and 36pl in Insausti et al., 1987). Area 36_pDM_ has been previously described also as area 36d (Suzuki and Amaral, 1994b) as it is located dorsal to area 36 of the perirhinal cortex and, although architectonically related to area 36r (rostral subdivision of the perirhinal cortex), it is distinct enough in terms of connections, thereby has received a different term. Our data is consistent with those divisions, and we have adopted the term area 36_pDM_ for consistency with the remaining areas of the TP (i.e., 38_DL_, 38_VL_). Area 36_pVM_ is followed caudally by 36r, which is mainly related with visual recognition memory (Meunier et al., 1993; Malkova et al., 2015).

#### Frontal Cortex

Architectonic divisions of the frontal cortex, including medial, orbitofrontal, and dorsolateral regions (Figure 2), used in this work were based on the descriptions made by Barbas and Pandya and colleagues (Barbas and Pandya, 1989; Pandya and Yeterian, 1990; Petrides and Pandya, 1999, 2002).

#### Insular Cortex

The nomenclature for the architectonic divisions of the insular cortex has been taken from Mesulam and Mufson (Morán et al., 1987) in *Macaca mulatta* (Figure 3).

### Injection Sites

From a total of 11 tracer injections included in this study, 3 were placed at different levels of area 38_DL_ of the temporal pole, another 3 in the 38_DL_/38_VL_ transition and 5 in 38_VL_. Size of injection site, location, and laminar involvement of the tracer injections are described in Table 1 and illustrated in Figure 1. Each case has been labeled by its number in the series of experiments followed by the tracer used.

**Table 1:** Location, laminar involvement, and rostrocaudal extent of the injection sites.

**Injection**	**Hemisphere**	**Case**	**Layers**	**Length (mm)**	**mm to TP^a^**	**mm to sts^b^**
38_DL_	Left	1FB_L_	I-V	2.3	0.5	2.5
	Right	7DY_R_	II-V	2.5	1.5	2.0
	Right	16DY_R_	I-III	2.0	0.5	2.5
38_DL_/38_VL_ border	Left	3FBL	I-V	2.5	0.5	1.5
	Right	7FB_R_	I-V	2.5	1.5	2.0
	Right	16FB_R_	I-IV	2.0	2.5	0.5
38_VL_	Left	2FBL	I-III	1.5	0.0	2.0
	Left	1DY_L_	I-V	1.0	1.0	2.5
	Right	6FB_R_	I-V	2.5	2.0	1.0
	Left	8DY_L_	I-V	1.0	2.5	2.5
	Left	14DY_L_	I-IV	2.5	0.0	3.5

a *Distance in mm from the rostral tip of the temporal pole (TP)*.

b *Distance from the rostral tip of the superior temporal sulcus (sts)*.

### Frontal Cortex

**Figure 4:**
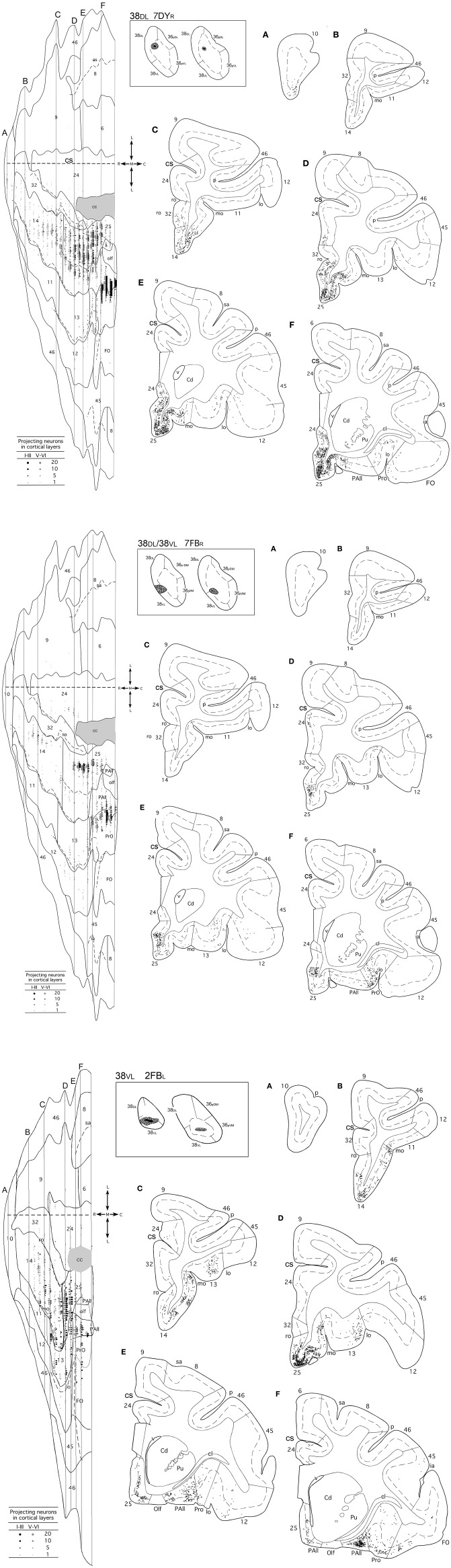
(made up of 3 panels). Unfolded map and coronal sections through the frontal cortex from rostral **(A)** to caudal **(F)** illustrate the distribution of retrogradely labeled neurons in representative cases with a DY injection in area 38DL (7DYR), FB injection in 38DL/38VL (7FBR), and a FB injection in area 38VL (2FBR). Each point corresponds to an individual neuron. Note that gray points in the map represent retrogradely labeled cells in layer III, whereas black points represent those found in layers V-VI. The density of retrograde labeling increases progressively from rostral to caudal and remains restricted primarily to the ventromedial frontal cortex. Within the orbitofrontal cortex, the highest density of retrograde labeling is in orbitofrontal areas 11, 13, PAll, and Pro. Note that the dorsolateral and dorsomedial prefrontal cortex contain practically no labeled neurons. In this case, the frontal cortex accounted for up to 19% of the total cerebral cortex input to area 38_DL_. Abbreviations as in previous figures: cs, cingulate sulcus; v, ventricle.

**Figure 5:**
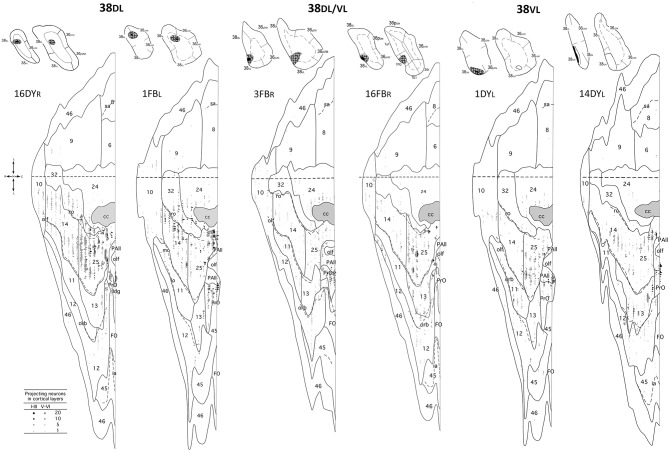
Unfolded maps of the frontal cortex show the density and topography of retrograde label distribution in two representative cases with injections in 38DL (16DYR, 1FBL), 38DL/38VL transition (3FB_R_, 16FB_R_), and in 38VL (1DYL, 14DYL). Abbreviations and conventions as in previous figures.

Overall, frontal cortex contributed with about 15–30% of the total cortical input to area 38_DL_ of the TP (30% in 16DY, 26% in 7DY, and 15% in 1FB, see Table 2). However, density of labeling decreased in cases with more ventral injections located at the 38_DL_/38_VL_ transition, which ranged from 4 to 8% in all the experiments, with a maximum of 15% in case 2FB (area 38_VL_). Coronal sections through the frontal lobe in Figure 4 and unfolded maps in Figure 5 illustrate the distribution of retrogradely labeled cells in specific architectonic areas and cortical layers.

**Table 2:** Percentage of labeled neurons in frontal cortex.

**Injection**	**Case**	**Orbitofrontal**					**Medial frontal**			**Dorsolateral**		**Dorsomedial**		**Caudal orbitofrontal**	
		**10**	**11**	**12**	**13**	**14**	**32**	**24**	**25**	**46**	**45**	**9**	**8**	**PAll**	**Pro**
38_DL_	1FB_L_	0.4^a^(3)^b^	0.1(1)	0.2(1)	0.3(3)	4(24)	2(8)	1(6)	7(42)	0	0	0.1(1)	0	0.1(0.3)	2(13)
	7DY_R_	0.2(1)	0.1(0.3)	1(3)	0.1(1)	2(7)	1(3)	1(2)	19(56)	0.2(1)	0.2(1)	0.1(0.1)	0.1(0.1)	6(16)	4(11)
	16DY_R_	1(2)	1(2)	1(1)	1(2)	4(11)	3(8)	1(3)	24(62)	0.1(0.2)	0	0.1(0.3)	0	2(4)	3(7)
38_DL_/38_VL_	3FB_R_	0.3(8)	0.2(5)	0.1(13)	0.1(9)	0.2(6)	0.1(2)	1(11)	1(15)	0.1(1)	0. (1)	0.1(1)	0.1(1)	1(25)	0.3(7)
	7FB_R_	0.1(0.1)	0	0.3(3)	1(4)	1(6)	0.1(1)	0.4(3)	6(43)	0	0.3(2)	0.1(0.1)	0	2(13)	3(25)
	16FB_R_	0.2(2)	1(3)	0.5(6)	1(9)	0.4(5)	1(2)	0	5(53)	0.1(0.1)	0	0.1(0.1)	0	1(17)	0.2(2)
38_VL_	6FB_R_	0.1(0.2)	1(6)	1(5)	2(8)	2(7)	0.1(0.4)	0.4(2)	7(30)	0.1(0.3)	0.1(0.3)	0.1(0.1)	0	8(37)	1(5)
	8DY_L_	0.1(2)	0.3(7)	1(20)	0.4(9)	0.5(10)	0.1(1)	0.2(4)	1(26)	0. (0.5)	0. (0.5)	0.2(4)	0.1(0.2)	0.6(13)	0.1(2.4)
	14DY_L_	0.1(2)	0.1(0.3)	0.8(11)	2(26)	0.6(8)	0.1(1)	0.13(2)	3(35)	0	0.1(1)	0	0	1(8)	0.41(6)
	1DY_L_	0.1(4)	1(7)	1(6)	0.1(2)	2(29)	0.1(2)	0.3(4)	2(27)	0.1(1)	0.1(1)	0.1(0.3)	0.1(0.1)	1.1(13)	1(6)
	2FB_L_	0	0.2(1)	0.1(1)	2(11)	5(29)	0.1(1)	0.1(1)	6(35)	0.1(0.1)	0. (0.2)	0.1(0.1)	0	3(17)	1(4)

a *Percentage of labeled neurons of total labeled neurons in the cerebral cortex*.

b *Percentage of labeled neurons of total labeled neurons in the frontal cortex*.

#### Medial Frontal Projection to Area 38_*DL*_

Area 25 contributed with 42–62% of the frontal projection to area 38_DL_. This was the heaviest projection in the whole cerebral cortex reported in this study (Table 2). Area 14 was the next highest with 7–24% of the frontal input to area 38_DL_ of the temporal pole. The subgenual portion of area 32 contributed with 3–8% of the frontal cortex input to area 38_DL_ of the temporal pole. Area 24 of the anterior cingulate cortex, and again specifically its subgenual portion, had 2–6% of the frontal label. As it can be appreciated in coronal sections (Figure 4A) and unfolded maps (Figure 5), retrograde labeling in these cases was primarily distributed in medial frontal areas 25 and 14. Area 10 of the frontal pole only contained 1–3% of the frontal labeled neurons.

#### Medial Frontal Projection to the 38_*DL*_/38_*VL*_ Transition and Area 38_*VL*_

Even though the highest density of retrograde label was still found primarily in the medial portion of areas 25 (15–53%) and 14 (5–29%) and less so in area 10 (0.1–8%; coronal sections in Figures 4B,C and unfolded maps in Figure 5), these densities were below those observed after more dorsal injections (area 38_DL_, see Table 2 and Figures 4A, 5). The density of retrograde labeling decreased substantially in the adjacent areas 32 and 24 of the medial frontal cortex with 0.4–2% and 0–4% of the labeled neurons in frontal cortex after more ventral injections in the lateral temporal pole.

Retrograde labeling took a general topography in medial frontal cortex, whereby injections at the 38_DL_/38_VL_ boundary resulted in a transitional pattern of retrograde labeling in frontal cortex. Like injections in 38_DL_, they labeled neurons primarily in infralimbic area 25 and in caudal orbitofrontal areas proisocortical (Pro) and periarchicortical (PAll), which represented 6, 3, and 2%, of the total cortical label, respectively (Figure 4B). However, and unlike dorsal injections, and more like ventral deposits in the lateral temporal pole, they labeled a higher density of cells in orbitofrontal cortex, especially in area 13. Although quantitatively light, this increase in density of labeled cells is noticeable in the unfolded maps (Figure 5). More ventral injections (in area 38_VL_) resulted in light density of labeling in areas 25, and 24 and 32 (i.e., 0.5% all together) and was distributed dorsal to the genu of the *corpus callosum*.

#### Orbitofrontal Projections to 38_*DL*_

The orbitofrontal projection to area 38_DL_ was numerically more modest than that from the medial frontal cortex. Nevertheless, the density of the orbitofrontal projection was still substantial at caudal level in periallocortical area PAll (0.3–16%) and proisocortical area Pro (7–13% of the frontal cortex labeling). Areas 12 and 13 of the orbitofrontal cortex contributed each with 1–3% of the frontal projection to the dorsolateral TP area 38_DL_, while area 11 with 0.3–2% (Table 2 and Figures 4, 5).

#### Orbitofrontal Projection to 38_*DL*_/38_*VL*_ and 38_*VL*_

As illustrated in Figures 4B,C, 5, injections in 38_DL_/38_VL_ transition and area 38_VL_ resulted in higher density of retrograde labeling in the caudal orbitofrontal cortex compared with more dorsal injections (i.e., in area 38_DL_). Area PAll accounted for 8–37% of the frontal projection to area 38_VL_, compared with 0.3–16% in cases with injections in 38_DL_. Area 13 had 2–26% of the frontal cortex labeled neurons, compared with 1–3% after injections in 38_DL_. Area 12 contributed with 1–20%, and area 11 of the medial orbitofrontal cortex contributed more modestly to the projection to area 38_VL_ (0.3–7%, Table 2).

In addition, retrograde labeling after ventrolateral injections was found more laterally in areas 14 and 25 than following injections in 38_DL_ (Figure 4). It is important to note that the density of retrogradely labeled neurons in areas 14 and 25 of the medial prefrontal cortex was still the highest of total frontal labeling (5–29% and 26–53%, respectively). However, unlike the frontal projection to 38_DL_, labeled neurons were primarily located in the orbitofrontal portion of areas 14 and 25.

#### Dorsolateral and Dorsomedial Prefrontal Cortex

In sharp contrast with the high density of labeling found in the medial and orbitofrontal cortices, dorsolateral and dorsomedial regions of the frontal cortex were characterized by an almost complete absence of labeling. Occasional retrograde labeled neurons were found in dorsomedial areas 8 and 9 (0–4%) and in dorsolateral areas 46 and 45 (Figures 4, 5 and Table 2). Only scattered retrogradely labeled cells were found in the dorsolateral portion of area 10 and the most rostral portion of the ventral bank of the principal sulcus, area 46, which contributed with only 0–1% of the total frontal cortical projection.

#### Laminar Distribution of Retrograde Labeling in Frontal Cortex

Injections in both areas 38_DL_ and 38_VL_ yielded a similar distribution of retrograde labeling in layers III and V-VI of area 25 (Figure 4). Areas 24, 32, and 10 had labeled neurons mainly in layer III. Retrograde labeling density decreased more rostrally in area 14, but still occupied layers III and V-VI. Retrogradely labeled neurons extended along layers III and VI of caudal orbitofrontal areas PAll and Pro, whereas in areas 11, 12, and 13 of the orbitofrontal cortex label was in layers III and V.

### Insular Cortex

**Figure 6:**
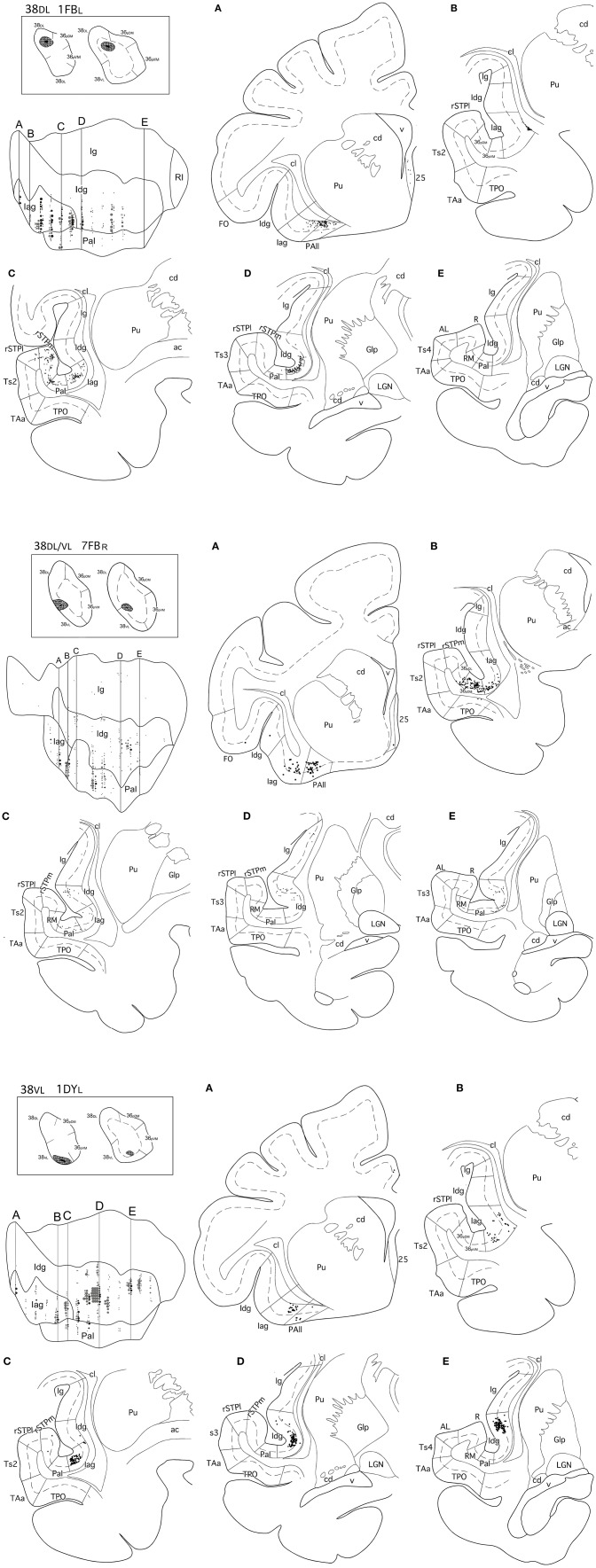
(made up of 3 panels). Unfolded map and coronal sections through the insula from rostral **(A)** to caudal **(E)** illustrate the distribution of retrogradely labeled neurons in representative cases with injections in area 38DL (1FBL), in 38DL/38VL (7FBR), and in area 38VL(1DYL). Abbreviations and conventions as in previous figures: ac, anterior commissure; Gp, *globus pallidus*; LGN, lateral geniculate nucleus; RI, rostrotemporal lateral auditory belt area; RM, rostrotemporal medial auditory belt area; rSTPl/rSTPm, rostral supratemporal areas lateral/medial.

**Figure 7:**
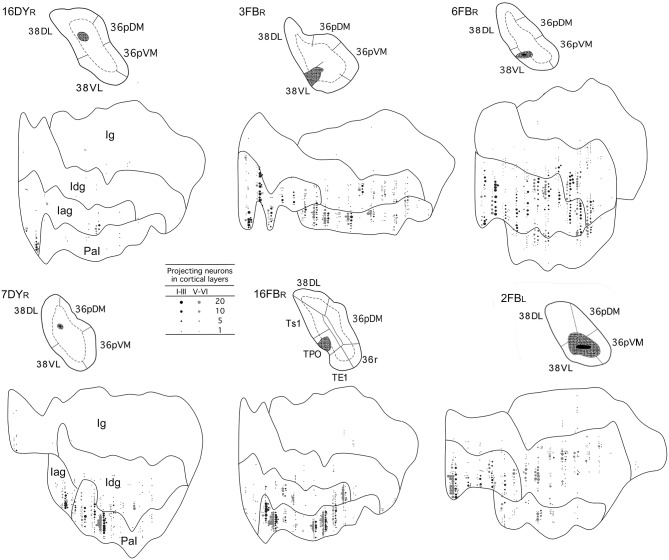
Two-dimensional unfolded maps of the insular cortex show the density and topography of the distribution of retrograde label in six representative cases with injections in 38_DL_ (16DY_R_, 7DY_R_), 38_DL/_38_VL_ transition (3FB_R_, 16FB_R_), and 38_VL_ (6FB_R_, 2FB_L_). Abbreviations and conventions as in previous figures.

The insula contained 5–10% of the total number of retrogradely labeled neurons in the cerebral cortex (Table 3). Within the insula, the highest density of retrograde labeling (i.e., 17–83%) originated in the agranular division (Iag), followed by the disgranular (Idg, 3–79%), and the parainsular (PaI, 3–63%) divisions (Figures 6, 7). The topographical distribution of the projection is described in the following paragraphs.

**Table 3:** Percentage of retrograde labeled neurons in the insula's architectonic areas (Iag, Idg, Ig, and PaI).

**Injection**	**Case**	**Iag**	**Idg**	**Ig**	**PaI**
38_DL_	1FB_L_	5^a^(70)^b^	1(22)	0	1(8)
	7DY_R_	1(29)	0.2(8)	0(0.1)	2(63)
	16DY_R_	1(83)	0(4)	0(4)	0.1(9)
38_DL_/38_VL_	3FB_R_	1(43)	0(14)	0(0.4)	1(43)
	7FB_R_	0.5(20)	0.8(34)	0(2)	1(44)
	16FB_R_	1(24)	0(3)	0.1(1)	3(73)
38_VL_	6FB_R_	3(67)	1(26)	0(1)	0.4(8)
	8DY_L_	2(34)	3(52)	0.1(1)	1(13)
	14DY_L_	4(41)	3(31)	0.4(3)	3(24)
	1DY_L_	1(17)	6(79)	0(1.2)	0(3)
	2FB_L_	2(43)	2(42)	0(7)	0(7)

a Percentage of labeled neurons of total labeled neurons in the cerebral cortex.

b *Percentage of labeled neurons of total labeled neurons in the insula*.

Retrogradely labeled neurons in the insular cortex showed a topographical arrangement (see coronal sections in Figure 6 and unfolded maps in Figures 6, 7). Injections in 38_DL_ resulted in high density of labeling mainly in the ventral portions of Iag and Idg, whereas injections located at the 38_DL_/38_VL_ transition labeled neurons progressively on more dorsal portions of Iag and Idg.

Cases with injections in 38_VL_ resulted in retrograde labeling that distributed ventrally in Iag, Idg, and PaI (Figures 6, 7). The granular insular cortex had only occasional labeled cells, especially in those cases with injections at the 38_DL_/38_VL_ transition, while ventral injections (38_VL_) resulted in a relatively higher density of labeled neurons in Ig (1–7%).

#### Laminar Distribution of Retrograde Labeling in the Insula

Within the rostral half of Iag, the density of labeled neurons was highest in layers V-VI (Figure 6). Here, labeled cells formed a continuous band that extended in depth up to the vicinity of the *claustrum*. However, in the caudal half of Iag, labeling also extended to layers II and III. Layers II-III and V of the rostral third of the dysgranular (Idg) division of the insular cortex had very low density of labeled cells. The parainsular cortex labeling was primarily in layers III and V-VI.

### Summary of the Projection

**Figure 8:**
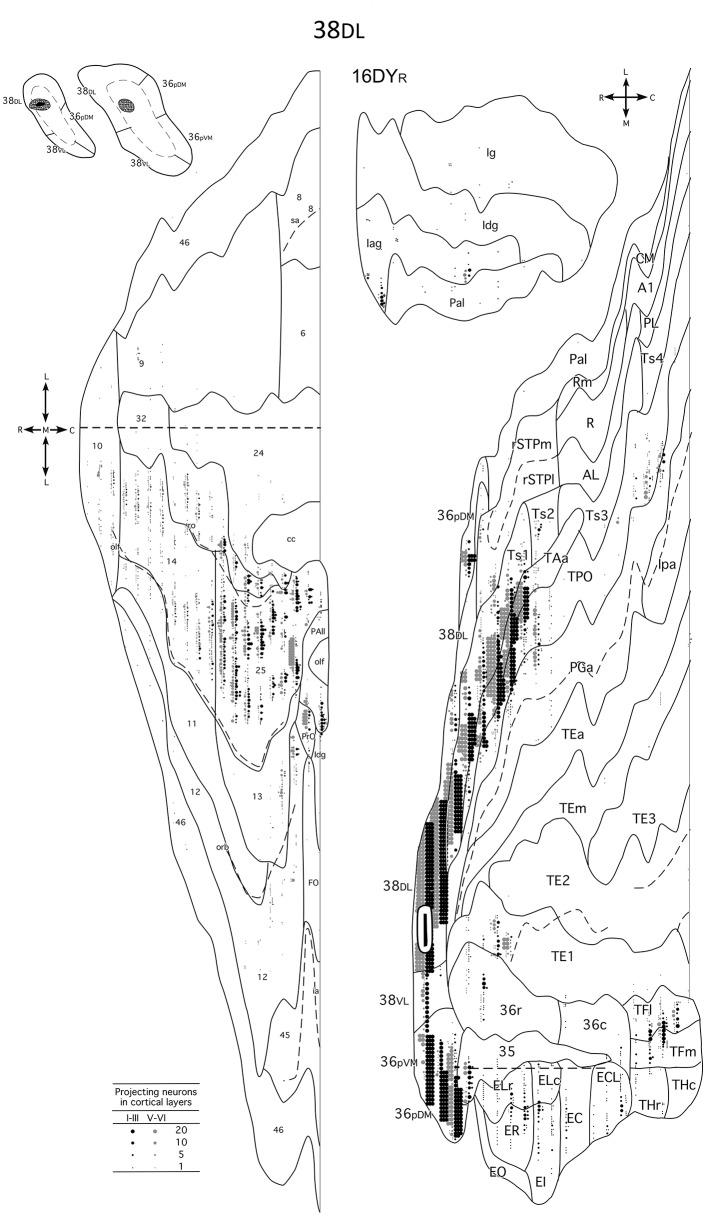
Unfolded maps of the temporal, frontal and insular projection to the dorsolateral TP in a representative case with an injection in area 38_DL_. Abbreviations and conventions as in previous figures: A1, auditory core area A1; CM, auditory cortex caudal medial area; E_C_, caudal subfield of the entorhinal cortex; E_CL_, caudal limiting subfield of the entorhinal cortex; E_I_, intermediate subfield of the entorhinal cortex; E_LC_, lateral caudal subfield of the entorhinal cortex; E_LR_, lateral rostral subfield of the entorhinal cortex; E_O_, olfactory dubfeld of the entorhinal cortex; E_R_, rostral subfueld of the entorhinal cortex; lpa, fundus of the superior temporal sulcus area Ipa; PGa, fundus of the superior temporal sulcus area PGa; PL, posterior lateal belt; Rm, rostrotemporal medial auditory belt area; R, rostral area of primary auditory cortex; TE1/2/3, anterior/middle/posterior area TE interior temporal gyrus area TE; TEa, interrior temporal gyrus TEa; TEm, inferior temporal gyrus area TEm; TFl, lateral portion of area TF; TFm, medial portion of area TF; THc, caudal portion of area TH; THr, rostral portion of area TH; Ts4, superior temporal gyrus area Ts4.

**Figure 9:**
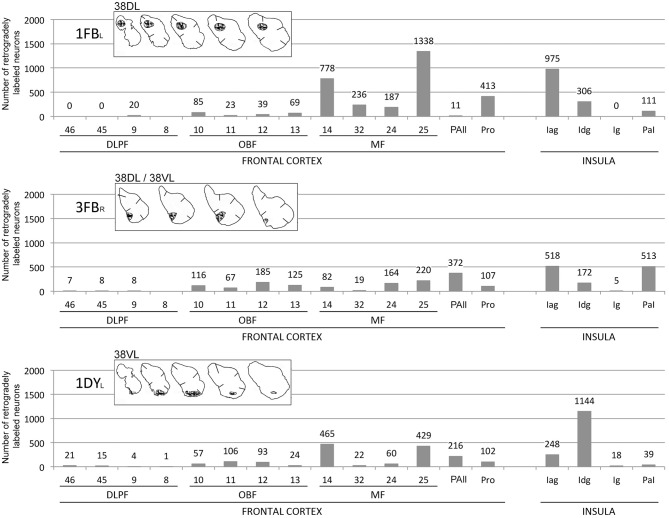
Histograms represent the total number of retrogradely labeled neurons in three representative cases with injections in 38_DL_ (1FB_L_), 38_DL/_38_VL_ (3FB_R_), and 38_VL_ (1DY_L_) in frontal and insular regions. Abbreviations as in previous figures: DLPF, dorsolateral prefrontal cortex; MF, medial frontal cortex; OBF, orbitofrontal cortex.

This study reports that around 50% of the total cortical input from frontal and insular cortices to the dorsolateral TP area 38_DL_ comes from: a) 25% medial frontal areas 24, 32, 14, 25; b) 15% orbitofrontal areas 11, 12, 13, Pro, PAll; and c) 10% insular areas Iag, Idg. Unfolded maps of the temporal, frontal, and insular cortices in Figure 8 illustrate the cortical projection to the lateral temporal pole. Histograms in Figure 9 with raw number of retrogradely labeled neurons provide an estimate of the contribution of each architectonic area to this projection.

#### Frontal Cortex

**Figure 10:**
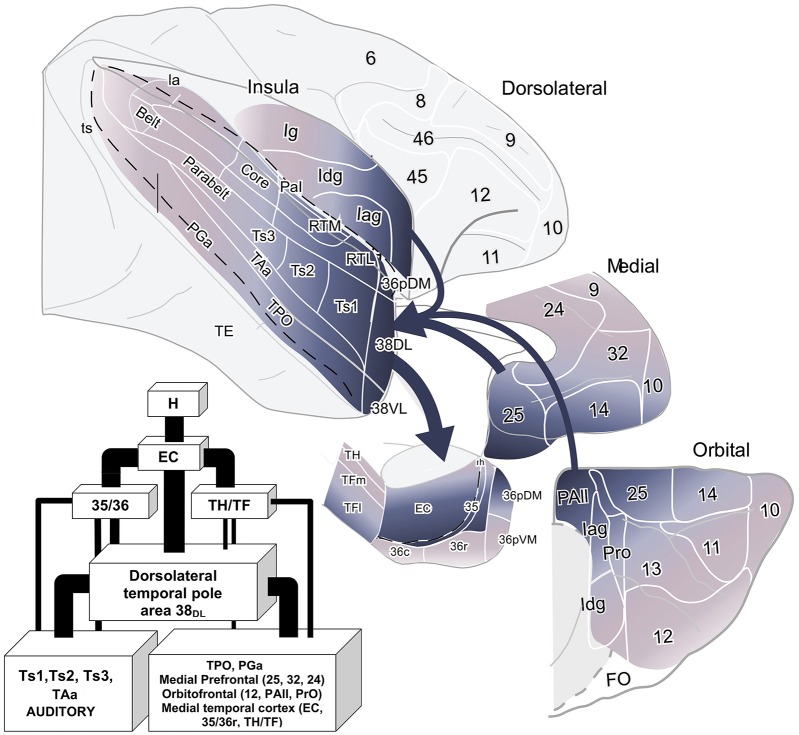
Schematic diagram of the cortical input to the dorsolateral TP area 38_DL_. The thickness of the arrows indicates the contribution in the projection. The flow diagram shows the anatomical pathway for auditory and multimodal memory. Abbreviations as in previous figures.

Overall, within the frontal cortex, the medial frontal and caudal orbitofrontal areas 14 and 25 contributed with the densest projection to the lateral TP areas. In contrast, the dorsolateral and dorsomedial prefrontal areas only contributed with a weak to almost inexistent projection to the lateral TP (Figure 10).

The frontal projection to the TP displayed a topographical disposition in which more medial regions of the prefrontal cortex send the densest projection to both areas of the lateral TP (25%, 38_DL_ and 38_VL_), with a heavier contribution to area 38_DL_. Although injections in ventrolateral TP area 38_VL_ labeled progressively more orbital-lateral portions of areas 14 and 25 and lateral orbitofrontal cortex area 13, the percentage of retrogradely labeled neurons was lower compared to medial areas 14 and 25.

Medial frontal cortex. In all the experiments, area 25 leaded the frontal projection to the TP with 15–62% of the frontal cortex labeling. The next highest was ventromedial frontal area 14, which contributes with 5–29% of the frontal projection to the temporal pole. The density of labeled cells decreased rostrally, so area 10 represented 0–8% of the frontal cortex input to the temporal pole. Labeling density also decreased in the prelimbic area 32 (0.4–8%) and anterior cingulate area 24 (1–11%).

Orbitofrontal cortex. Within the orbitofrontal cortex, the highest density of the projection to the TP originated in the areas located immediately rostral to the insular cortex, i.e., areas PAll (0.3–37%) and Pro (2–25%) followed by areas 13 (1–26% and 12 (1–20%). Only scattered labeled neurons were found in the orbital part of the frontal pole of areas 10 and area 11. The projection continued caudally in the orbital portion of areas 14 and 25 (Figures 4, 5).

Dorsolateral and dorsomedial prefrontal cortex. This projection was virtually absent, with only scattered labeled cells primarily in areas 10, 9, and the ventral bank of the principal sulcus (area 46).

#### Insular Cortex

The insular cortex contributed about 10% of the total cortical input to area 38_DL_. The ventral part of the agranular insula originated the bulk of the projection to the dorsolateral TP area 38_DL_. The topography of this projection was as follows: more ventral portions of Iag and Idg send projections to the dorsolateral TP area 38_DL_, while the more dorsal portions of Iag and Idg, together with a contribution from Ig, send projections progressively to more ventral TP areas 38_DL/VL_ and 38_VL_.

## Discussion

The present study provides, for the first time, a comprehensive topographical and quantitative description of the frontal and insular cortex projection to area 38_DL_ of the dorsolateral temporal pole (TP, see Figures 8 and 10). This study reports that around 50% of the cortical input to area 38_DL_ comes from the frontal and insular cortices, specifically in: a) medial frontal areas 14, 25, 24, and 32 (25%); b) orbitofrontal areas 11, 12, 13, Pro, and PAll (15%); and c) insular areas PaI, Iag, and Idg (10%). These findings build upon and complement the previous study on the temporal afferents to the lateral TP (Muñoz-López et al., 2015), in which we showed that the remainder 50% of the cortical input to area 38_DL_ comes from within the temporal lobe in: a) rostral superior temporal gyrus higher order processing auditory areas Ts1 and Ts2, and TAa (30%); b) polysensory area of the dorsal bank of the superior temporal sulcus (area TPO, 10%); and c) medial temporal cortex (10%). This now provides a bigger picture of the quantitative inputs to area 38_DL_.

We discuss these results in relation with previous studies and focus on their potential relevance for auditory memory as part of a wider limbic circuitry underlying memory processing. Finally, we set the results of this study in the context of human imaging and patient work.

### Cortical Input to the Temporal Pole

Our findings confirm those from previous studies (Jones and Powell, 1970; Mesulam and Mufson, 1982; Mufson and Mesulam, 1982; Markowitsch et al., 1985; Morán et al., 1987; Kondo et al., 2003; Saleem et al., 2008) and add that only about one third of the dorsolateral temporal pole's input arises in auditory processing areas, but up to 70% of its input originates in areas beyond these auditory processing regions. These areas include polysensory temporal cortex, ventral insula, caudal orbitofrontal and medial frontal cortices, i.e., the more limbic-like subdivisions of the frontal and insular cortices. A functional implication of this new finding is that, beyond being part of an auditory pathway, there is the possibility that the dorsolateral TP forms part of a wider circuitry underlying memory processing in the auditory domain, amongst other functions.

Based on structural and functional MRI connectivity data of the TP in humans (Fan et al., 2014; Pascual et al., 2015), one may expect other cortical sources of input to the dorsolateral TP apart from temporal, frontal, and insular. However, we have not observed projections from any other cortical area despite having analyzed the whole of the cerebral cortex in all cases, except for the occipital pole. In fact, up to this date, there have not been reports on projections from other cortical areas other than the ones reported in this study even when larger tracer injections have been used (Markowitsch et al., 1985). It is not the case that the tracers used in this or previous studies lack sensitivity to distant projections. For example, Markowitsch's study reported distant connections of the TP with the pulvinar and the brain stem, however no other cortical areas except for frontal, insular or temporal cortices. In our study, we confirmed the thalamic connections as far as the caudomedial pulvinar (data not shown here), but none from other cortical areas.

### The Temporal Pole and the Ventromedial Frontal Cortex

The limbic circuit that underlies memory processing in monkeys includes the hippocampal formation, the parahippocampal region, the diencephalon (anterior thalamic and mediodorsal thalamic nuclei), the ventromedial frontal cortex including the caudal orbitofrontal and medial frontal cortex (Aggleton and Mishkin, 1983a,b; Bachevalier and Mishkin, 1986; Xiang and Brown, 2004; Vertes et al., 2015) see a recent review in Bubb et al. (2017). The data presented in this study provides data on the contribution of the dorsolateral TP to this wider limbic memory processing pathway (Figure 10).

To understand the functional organization of the TP connections with all these memory related areas, we need to take at least three additional issues into account. First, the lesion of the ventromedial frontal cortex damages delayed memory in a similar way as other structures that form part the limbic circuit, such as the medial diencephalon or the medial temporal lobe (Bachevalier and Mishkin, 1986). Second, neurons in ventromedial frontal cortex show memory related responses, especially in the medial frontal cortex (Xiang and Brown, 2004). Therefore, this points to the ventromedial frontal cortex as part of the limbic circuitry for memory (Bachevalier and Mishkin, 1986; Xiang and Brown, 2004), an idea supported by many studies thereafter (see review in Bubb et al., 2017). Second, medial temporal lobe removals leading to memory problems in both visual and auditory memory disconnect both inferotemporal cortex area TE and rSTG from the mediodorsal thalamus and ventromedial frontal cortex (Baxter et al., 1998; Goulet et al., 1998; Muñoz et al., 2009). This medial temporal-diencephalic-frontal disconnection has been put forward as one of the reasons that, at least in part, explains the memory impairment seen after medial temporal damage (see Muñoz et al., 2009 for more details on this discussion). Third, the importance of the ventromedial frontal cortex in memory in humans has been highlighted in fMRI studies (Takashima et al., 2007; Euston et al., 2012; de la Vega et al., 2016). However, the functional organization of the ventromedial frontal cortex calls for further research, in part because the contribution of the diverse anatomical divisions of the medial frontal cortex and their role in cognition remains unclear (Córcoles-Parada et al., 2017).

In sum, the results here point to the possibility that the dorsolateral TP forms part of an auditory memory pathway embedded within a wider limbic circuit that might contribute among other functions to multimodal memory processing.

### The Temporal Pole and the Insula

Our results agree with previous anatomical reports (Mesulam and Mufson, 1982; Mufson and Mesulam, 1982; Morecraft and Van Hoesen, 1992; Morecraft et al., 1992, 2015), and confirm that the more visceral-related areas of the insula, i.e., parainsular, agranular and disgranular, contribute with 10% of the cortical projections to the dorsolateral temporal pole, whereas the dysgranular and granular sectors send progressively lighter projections.

The parainsular, agranular and dysgranular divisions of the ventral insula, considered paralimbic, have direct and reciprocal connections with the entorhinal (Insausti et al., 1987), perirhinal, and posterior parahippocampal cortices (Suzuki and Amaral, 1994b; Lavenex et al., 2002). Collectively these findings and our new results indicate that the ventral insula's cortical network overlaps substantially with that of the dorsolateral temporal pole, especially the part of the network involved in memory processing.

However, the insular cortex is a complex structure involved in more than just one network (Evrard, 2018). For example, the parainsular, agranular and dysgranular divisions of the insula also have direct connections with the hypothalamic and brain stem nuclei that control autonomic (visceral) functions, especially associated with the olfactory, gustatory, gastrointestinal tract, blood pressure, and heart rate (Nieuwenhuys et al., 2012; Aleksandrov and Aleksandrova, 2015; Evrard, 2018). In addition, these same divisions of the insula are strongly connected with the amygdala (Mufson et al., 1981; Stefanacci and Amaral, 2000) and are important for affiliative processing (Jezzini et al., 2015). In contrast, the areas of the insula that do not project to the dorsolateral temporal pole, i.e., the dorsal and posterior divisions of the insula (granular and dysgranular), are primarily involved in somato-sensory and pre-motor functions (Morecraft and Van Hoesen, 1992; Morecraft et al., 1992, 2015), amongst other functions (Friedman et al., 1986; see review in Evrard, 2018).

Therefore, although, the role of the ventral agranular insula in memory is still unknown, the anatomical data on its connectivity with the TP and medial temporal cortex points to its involvement in the limbic circuitry for memory processing. In addition, the ventral insula may be a node in the limbic memory system linking it with the autonomic nervous system.

### Functional Implications for Human Studies

Lesion, physiological, imaging and patient studies, especially with humans, can shed a more direct light on function. This anatomical study with primates provides information on the structure of the dorsolateral TP network/s and an indication on function. If we take a speculative view, we can adventure some brief functional implications for human research.

Early lesions studies of the TP in primates pointed to its involvement in affiliative, social, and emotional behavior (Myers, 1970, 1972; Kling and Steklis, 1976; Kling et al., 1993). In primates, the TP has dense connections with the amygdala, so strong that they form a distinct fasciculus (Klingler and Gloor, 1960). These connections support the involvement of the TP in emotion. In the same vein, an increasing number of functional neuroimaging experiments in humans show activation of the TP in a wide variety of tasks involving the processing of social and emotional stimuli (Baron-Cohen et al., 1999; Beauregard et al., 2001; Tillfors et al., 2001) as well as semantic cognition (Lambon Ralph et al., 2017). The involvement of the TP in semantic cognition has been supported by the striking temporopolar degenerative disorder characteristic of semantic dementia (Mummery et al., 2000; Hodges and Patterson, 2007; Lambon Ralph and Patterson, 2008; Acosta-Cabronero et al., 2011; Lambon Ralph et al., 2017).

A key finding is that our results here are consistent with human structural, fMRI network analysis and patient data in that, based on connectivity, they point to the dorsolateral TP as part of the limbic circuitry for memory integrating auditory with multimodal and higher order multimodal information. Furthermore, the dorsolateral TP may also be part of networks important for social, emotional and semantic cognition. The present quantitative study of the connectivity of the TP in non-human primates contributes an anatomical foundation for these ideas and calls for further research on the functional organization of the TP networks.

## Conclusion

The present quantitative study, collectively with previous behavioral, physiological and lesion studies in primates, point to the possibility that the dorsolateral temporal pole forms part of a wider limbic circuit that might contribute among other functions to multimodal memory processing.

## Data Availability Statement

The datasets generated for this study are available on request to the corresponding author.

## Ethics Statement

Experiments were carried out in strict adherence to the Guide for Care and Use of Laboratory Animals and under approved NIMH Animal Study Proposal and the European Union rules for care and use of animals (UE 86/609/CEE), and the supervision and approval of the Ethical Committee of Animal Research of the University of Castilla-La Mancha, Spain.

## Author Contributions

MC-P helped writing the manuscript and preparing figures and references. MU-M helped in preparing the manuscript, the figures, and interpreting the results. RM contributed in the discussion of the results and writing this manuscript. RI contributed in the discussion of the results and writing this manuscript. MM helped design the study and mentored MM-L during her work. MM-L designed the study, conducted the experiments, processed the tissue, analyzed the data, created the figures, and supervised the writing of this manuscript, and preparation of the figures.

### Conflict of Interest

The authors declare that the research was conducted in the absence of any commercial or financial relationships that could be construed as a potential conflict of interest.

## Abbreviations

36_pDM_: Dorsal medial divisions of the temporal pole
36_pVM_: Ventral medial divisions of the temporal pole
38_DL_: Dorsal lateral division of the temporal pole
38_VL_: Ventral lateral division of the temporal pole
DY: Diamidino Yellow
EC: Entorhinal cortex
FB: Fast Blue
Iag: Agranular division of the insula
Idg: Disgranular division of the insula
Ig: Granular division of the insula
PaI: Parainsular cortex
PAll: Frontal periallocortical area (Barbas 1992)
Pro: Frontal proisocortical area (Barbas 1992)
rSTG: Rostral superior temporal gyrus
TAa: Superior temporal gyrus area TAa (Seltzer and Pandya, 1978 1989)
TE1/TE2/Tem: Inferior temporal gyrus areas TE1/TE2/TEm (Von Bonin and Bailey 1947)
TF: Medial temporal cortical area TF (Von Bonin and Bailey 1947)
TH: Medial temporal cortical area TH (Von Bonin and Bailey 1947)
TP: Temporal pole
TPO: Superior temporal gyrus area TPO (Seltzer and Pandya, 1978 1989)
Ts1/Ts2/Ts3: Superior temporal gyrus area Ts1/Ts2/Ts3 (Seltzer and Pandya, 1978 1989).
